# TACE-HAIC versus HAIC combined with TKIs and ICIs for hepatocellular carcinoma with a high tumor burden—a propensity-score matching comparative study

**DOI:** 10.3389/fimmu.2025.1664756

**Published:** 2025-11-24

**Authors:** Chunxue Wu, Yunlong Dong, Xinge Li, Wenbo Shao, Guangshun Wang, Huiyong Wu, Xu Chang

**Affiliations:** 1 Shandong Cancer Hospital and Institute, Shandong First Medical University and Shandong Academy of Medical Sciences, Jinan, China; 2 Department of Thoracic Surgery, Tianjin Baodi Hospital, Baodi Hospital Affiliated to Tianjin Medical University, Tianjin, China; 3 Central Hospital Affiliated to Shandong First Medical University, Jinan, China

**Keywords:** hepatocellular carcinoma, transarterial chemoembolization (TACE), hepatic arterial infusion chemotherapy (HAIC), combination therapy, high tumor burden

## Abstract

**Purpose:**

The present study aimed to comparatively examine transarterial chemoembolization (TACE) plus hepatic arterial infusion chemotherapy (HAIC) in combination with tyrosine kinase inhibitors (TKIs) and immune checkpoint inhibitors (ICIs) versus HAIC alone in combination with TKIs and ICIs for efficacy and safety in individuals with high tumor burden (major portal vein tumor thrombosis [PVTT] Vp3–4 or/and tumors larger than 10 cm) hepatocellular carcinoma (HCC).

**Methods:**

Totally 363 inoperable HCC cases with high tumor burden administered TACE-HAIC plus TKI and ICI (TACE-HAIC combination group, n=119) or HAIC plus TKI and ICI (HAIC combination group, n=244) were recruited between October 2020 and January 2024, and propensity score matching (PSM) was utilized for matching patients. Overall survival (OS), progression-free survival (PFS), objective response (ORR), disease control (DCR) rates, and safety signals were assessed.

**Results:**

Following PSM (1:2), 87 cases in the TACE-HAIC combination group were matched to 143 cases in the HAIC combination group. Median OS (26.8 vs. 19.1 months, *p* = 0.233) and PFS (11.17 vs. 9.01 months, *p* = 0.133) were similar in the TACE-HAIC and HAIC combination groups. ORRs were 58.0% and 64.4% in the HAIC and TACE-HAIC combination groups, respectively (*p* = 0.341). DCR were 90.9% and 94.3% for these groups, respectively (*p* = 0.360). Both univariate and multivariate analyses revealed no differences between the two groups pre- and post-matching. The commonest adverse events (AEs) included thrombocytopenia, hypertension, and increased AST (aspartate aminotransferase) and ALT (alanine aminotransferase) of any grade pre- and post-PSM.

**Conclusions:**

For HCC patients with high tumor burden, HAIC demonstrates comparable efficacy to TACE-HAIC both in combination with TKIs and ICIs. Therefore, HAIC should be the preferred local therapeutic strategy over TACE-HAIC in HCC patients with high tumor burden.

## Graphical Abstract

**Figure:**
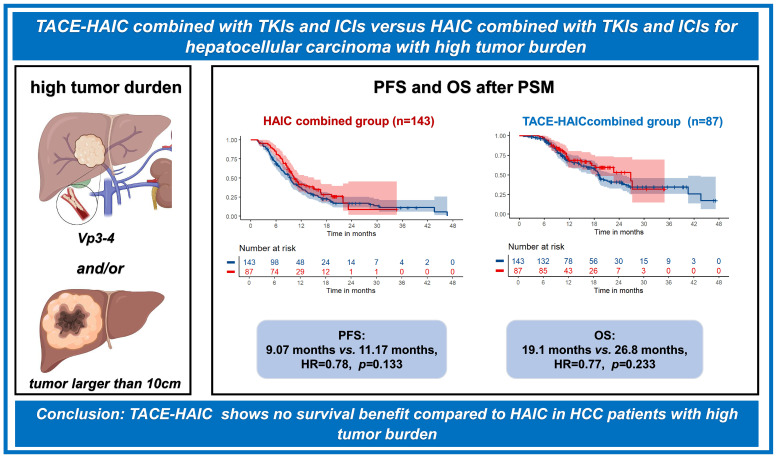

## Introduction

Hepatocellular carcinoma (HCC) ranks as the sixth most prevalent cancer and the third leading cause of cancer-related mortality globally (1, 2). Approximately 72% of all HCC cases are detected in Asian countries (3), with China comprising >50% (3). The majority of HCC diagnoses occur at advanced disease stages because of the insidious onset of this malignancy, when curative surgical resection is infeasible, leading to a meager 5-year survival rate of 10-18% (4, 5). HCC cases commonly have a high tumor burden (tumor larger than 10 cm, major portal vein tumor thrombosis (PVTT) Vp3-4) often manifesting as prevalent features (6). Such high-tumor-burden profiles are particularly common in Chinese patients, contributing to their extremely poor prognosis (3).

The development of systemic therapies has brought new hope for advanced HCC cases. Currently, atezolizumab + bevacizumab is advocated as the first-line therapeutic regimen in unresectable advanced HCC (7). Additionally, clinical trials, e.g., KEYNOTE-524 and CARES-310, have revealed that using tyrosine kinase (TKIs) and immune checkpoint (ICIs) inhibitors in combination has promising efficacy in advanced HCC (8, 9). However, patients with high tumor burden continue to experience suboptimal outcomes from the above therapies, attributed to their intrinsically unfavorable survival prognosis. This emphasizes the urgency to investigate more effective combinations of treatment strategies.

As demonstrated previously by our team, hepatic arterial infusion chemotherapy (HAIC) applied in combination with lenvatinib and ICIs represents a safe and effective treatment approach in HCC cases showing high tumor burden, demonstrating substantially improved overall survival (OS), progression-free survival (PFS), and objective response rate (ORR) versus lenvatinib and PD-1 inhibitors, with tolerable toxicity (10–12). Recently, an interesting study reported atezolizumab + bevacizumab in combination with TACE-HAIC demonstrated promising outcomes, with a median PFS of 10.1 months (95% confidence interval [CI]: 8.4-NA) and the median OS still pending. At one year, OS and PFS rates were 92.8% (95%CI: 86.1-100.0) and 42.9% (95%CI: 31.3-58.7), respectively (13). TACE-HAIC is presently considered a relatively new local treatment modality, which reduces resistance to HAIC and prolongs patient survival (14–21). However, most studies on TACE-HAIC are single-arm or use TACE for comparison, with a direct comparison between TACE-HAIC and HAIC lacking. In light of the potential for improvement in HCC cases with high tumor burden, the present study was designed to compare the efficacy and safety of TACE-HAIC in combination with TKIs and ICIs to HAIC in combination with TKIs and ICIs, to determine a more suitable local therapeutic strategy.

## Methods

Between October 2020 and January 2024, 363 inoperable HCC cases with high tumor burden were enrolled. HCC diagnosis was based on non-invasive methods or biopsy confirmation. Inclusion criteria were: (1) age of 18–80 years; (2) liver function as Child-Pugh class A or B; (3) Eastern Cooperative Oncology Group (ECOG) performance status of 0-2; (4) confirmation of Vp3–4 or/and tumor diameter ≥10 cm; (5) measurable liver tumor(s) per response evaluation criteria in solid tumors 1.1 (RECIST 1.1); (6) no prior systemic therapy. Exclusion criteria were: other concurrent malignancies, incomplete patient records, or loss to follow-up.

The present single-center retrospective study was approved by the Ethics Committee of our institution (SDTHEC 202410032). Written informed consent for the use of clinical data in research was obtained from all participants at the time of treatment. Data were de-identified by removing personal identifiers, following the Declaration of Helsinki 1955.

### Treatment protocol

Oral TKIs were initiated 3–7 days before treatment initiation for tolerance assessment. TKIs included sorafenib, lenvatinib, apatinib, and donafenib. Doses were adjusted for toxic effects as necessary. All TKIs were administered per the package inserts. Dose adjustment was performed according to the package insert when clinically necessary. PD-1 inhibitors such as sintilimab, camrelizumab, toripalimab, and tislelizumab were administered strictly in accordance with the respective specifications.

For HAIC, a microcatheter was positioned into the primary feeding hepatic artery for administering FOLFOX-based chemotherapy: oxaliplatin (85 mg/m^2^ for 0 to 2 h) on day 1, calcium folinate (400 mg/m^2^ for 2 to 3 h) on day 1, and fluorouracil (400 mg/m^2^ as a bolus at 3 h) on day 1, followed by 2400 mg/m^2^ over 46 h on days 1-2. After the initial HAIC procedure, the catheter and sheath were removed. Re-catheterization was performed for subsequent cycles. Maintenance therapy with TKIs and ICIs was continued thereafter. HAIC was conducted at 3–4-week intervals as needed.

TACE-HAIC was carried out as reported previously (22, 23). In brief, chemoembolization was conducted with 30 mg/m^2^ of epirubicin, lobaplatin 50 mg or raltitrexed added to 2–5 mL of lipiodol (10ml, 895 RMB). Next, up to 20 mL of lipiodol was administered by injection into the tumor-feeding artery until blood stasis in the target artery. The dose of chemoembolization lipiodol was based on the patient’s liver function, tumor size, vascularity, and body surface area. Additionally, gelatin sponge (Gelfoam150, 1050 RMB) particles were utilized if necessary. If an artery–portal vein fistula was performed, it was occluded before mixture embolization by a spiral steel ring based on angiography images and the doctor’s experience. Then, a catheter was positioned in the tumor-feeding artery, followed by fixation for the infusion of FOLFOX-based chemotherapeutics as the HAIC procedure. Repeated TACE-HAIC was conducted at 3–4-week intervals. The definitions of HAIC combination and TACE-HAIC combination groups are found in Supplement Pages 1-4, **Supplementary Figures S1–S4**.

### Follow−up and response assessment

Treatment responses were examined by two investigators, followed by verification by an independent radiologist. The primary tumor response was assessed employing RECIST 1.1. The anticancer effect was evaluated by OS, PFS, ORR, and disease control rate (DCR). OS was defined as the duration from initial HAIC therapy to death or the final follow-up, while PFS was the interval from initial HAIC therapy to disease progression or final follow-up. ORR was the rate of cases with complete (CR) or partial (PR) response, and DCR represented the percentage of cases with CR, PR, or stable disease (SD).

Adverse effects linked to treatment were recorded, e.g., hematotoxic and gastrointestinal effects, liver function changes, and immune-associated events.

### Statistical analysis

To minimize confounders and decrease selection bias, propensity score matching (PSM) was conducted, using 1:2 nearest-neighbor matching with no replacement, employing a caliper width of 0.05. Propensity scores were obtained by logistic regression utilizing covariates such as sex, age (<60 vs. ≥60), hepatitis B virus (HBV) infection (absent vs. present), tumor size (<10 cm vs. ≥10 cm), Child-Pugh grade (A vs. B), BCLC stage (A/B vs. C), extrahepatic spread (absent vs. present), Vp3-4 (absent vs. present), and tumor number (single vs. multiple).

Kaplan-Meier curve analysis was performed for survival outcomes, with the log-rank test utilized to compare groups. Univariable and multivariable analyses were conducted by Cox regression. Data were analyzed using R version 4.2.2 (R Foundation for Statistical Computing, Austria). Two-sided P<0.05 reflected statistical significance.

## Results

### Patient features

**Figure 1:**
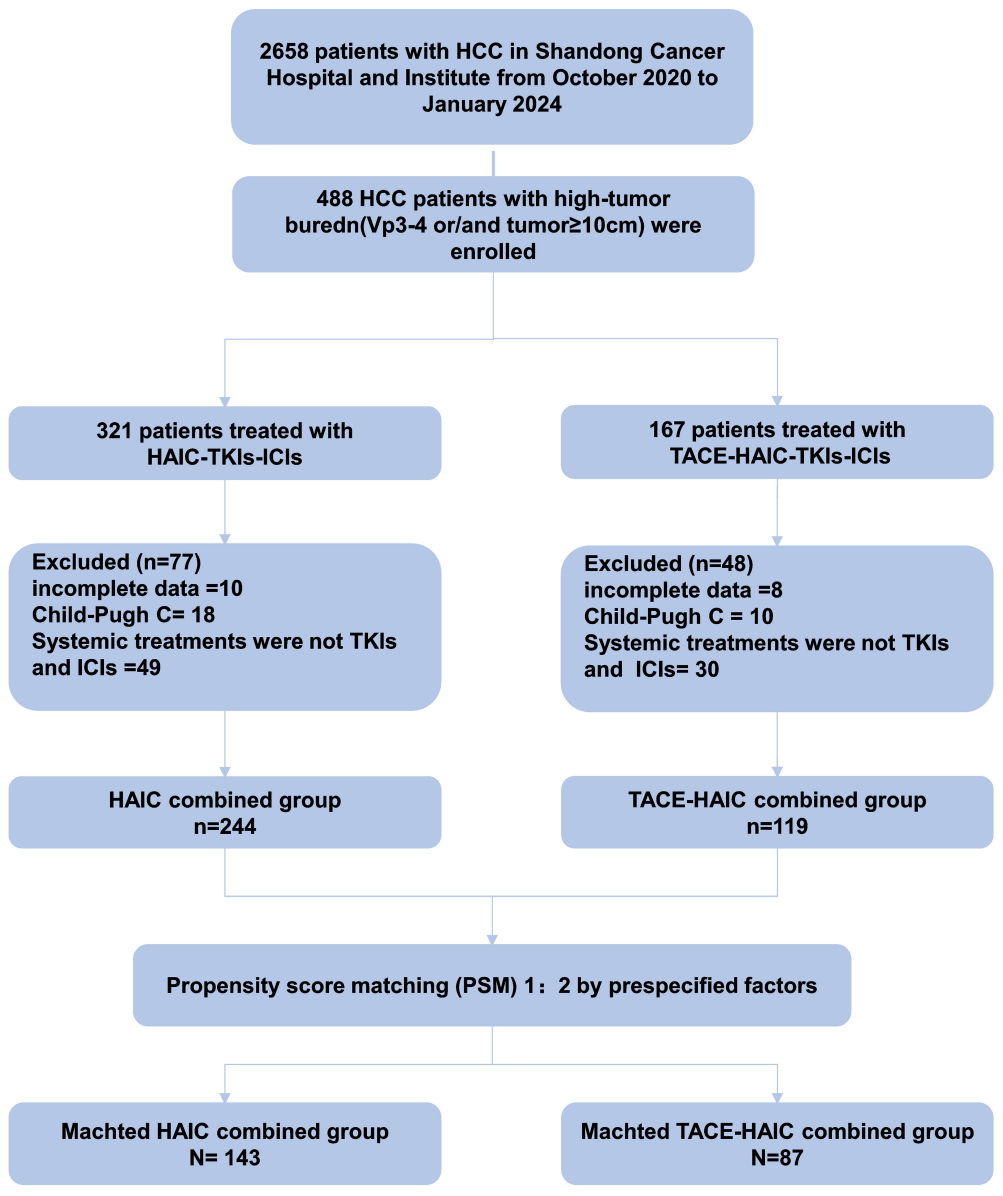
Patient selection flowchart.

Totally 363 patients were enrolled in *xxxx* Hospital, of whom 119 were administered TACE-HAIC plus TKI and PD-1 (TACE-HAIC combination group) and 244 received HAIC plus TKI and PD-1 (HAIC combination group). **Figure 1** depicts the study flowchart. Before PSM, the HAIC combination group had more patients at the BCLC C stage (97.5% vs. 89.1%, *p* = 0.002) and more cases with VP3-4 (95.1% vs. 67.2%, *p* < 0.001). Meanwhile, the TACE-HAIC combination group included more patients with a tumor diameter over 10 cm (79.8% vs. 55.3%, *p* < 0.001) and more cases with multiple lesions (82.4% vs. 67.6%, *p* = 0.005). After PSM (1:2), 87 cases in the TACE-HAIC combination group were matched to 143 in the HAIC combination group. Baseline features were comparable post-PSM in both groups (all *p*>0.05; **Table 1** and **Supplementary Figures S5, S6**).

**Table 1:** Baseline characteristics before and after PSM.

Characteristics	Before PSM			After PSM		
	HAIC combined N=244(%)	TACE-HAIC combined N=119(%)	*P*	HAIC combined N=143(%)	TACE-HAIC combined N=87(%)	*P*
Age (years)			0.065			0.263
<60 ≥60	184(75.4) 60(24.6)	78(65.5) 41(34.5)		108(75.5) 35(24.5)	59(67.8) 28(32.2)	
Gender			0.167			0.877
Male	224(91.8)	103(86.6)		132(92.3)	79(90.8)	
Female	20(8.2)	16(13.4)		11(7.7)	8(9.2)	
Etiology			0.796			0.717
Hepatitis B virus	219(93.6)	105(88.2)		130(90.9)	77(88.5)	
Others	25(6.4)	14(11.8)		13(9.1)	10(11.5)	
BCLC stage			0.002			0.686
A/ B	6(2.5)	13(10.9)		3(2.1)	4(4.6)	
C	238(97.5)	106(89.1)		140(97.9)	83(95.4)	
Tumor diameter (cm)			<0.001			0.889
<10	109(44.7)	24(20.2)		42(29.4)	24(27.6)	
≥10	135(55.3)	95(79.8)		101(70.6)	63(72.4)	
AFP (ng/ml)			0.510			0.773
<400	88(36.1)	38(31.9)		50(35.0)	28(32.2)	
≥400	156(63.9)	81(68.1)		93(65.0)	59(67.8)	
Tumor number			0.005			0.925
Single	79(32.4)	21(17.6)		30(21.0)	17(19.5)	
Multiple	165(67.6)	98(82.4)		113(79.0)	70(80.5)	
Child-Pugh class			0.012			0.642
A	218(89.3)	94(79.0)		126(88.1)	74(85.1)	
B	26(10.7)	25(21.0)		17(11.9)	13(14.9)	
VP classification (PVTT)			<0.001			0.350
VP3-4	232(95.1)	80(67.2)		133(93.0)	77(88.5)	
others	12(4.9)	39(32.8)		10(7.0)	10(11.5)	
Extrahepatic spread			1.000			0.546
Present	96(39.3)	47(39.5)		63(44.1)	34(39.1)	
Absent	148(60.7)	72(60.5)		80(55.9)	53(60.9)	
Tyrosine Kinase Inhibitors			0.056			0.277
sorafenib	30(12.3)	12(10.1)		20(14.0)	10(11.5)	
lenvatinib	139(57.0)	83(69.7)		82(57.3)	59(67.8)	
apatinib	75(30.7)	24(20.2)		41(28.7)	18(20.7)	
Immune checkpoint inhibitors			0.902			0.702
camrelizumab	129(5.9)	66(55.5)		73(51.0)	52(59.8)	
sintilimab	44(18.0)	23(19.3)		32(22.4)	17(19.5)	
tislelizumab	47(19.3)	22(18.5)		24(16.8)	13(14.9)	
toripalimab	15(6.1)	4(3.4)		7(4.9)	3(3.5)	
othres	9(3.7)	4(3.4)		7(4.9)	2(2.3)	
Totalbilirubin (mmol/L, mean±SD)	20.3±14.7	20.9±11.6	0.714	20.7±12.4	20.5±10.8	0.884
Albumin (g/L, mean±SD )	40.4±5.1	38.5±5.3	0.009	39.5±5.3	39.3±4.6	0.757

PSM, Propensity Score Matching; HAIC, Hepatic Arterial Infusion Chemotherapy; TACE, Transarterial Chemoembolization; BCLC, Barcelona Clinic Liver Cancer; AFP, Alpha-fetoprotein; PVTT, Portal Vein Tumor Thrombus.

### Efficacy

#### Before PSM

**Figure 2:**
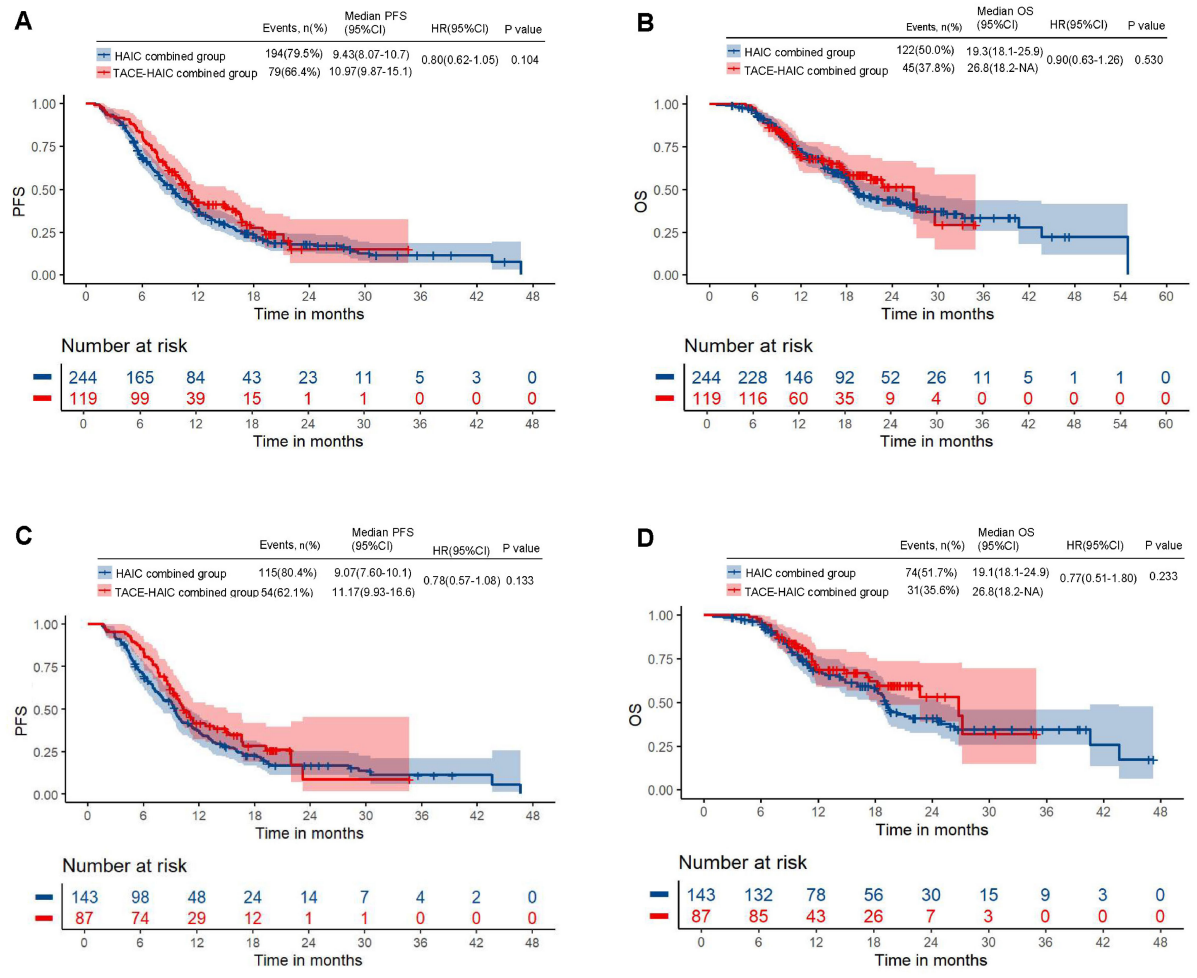
Kaplan–Meier curves of progression-free survival and overall survival before **(A, B)** and after **(C, D)** matching.

Median follow-up times were 23.63 (95%CI: 20.78-26.48) months and 17.60 (95%CI: 14.85-20.35) months in the HAIC combination and TACE-HAIC combination groups, respectively (*p* < 0.001). Totally 45 (37.8%) of 119 cases in the TACE-HAIC combination group and 122 (50.0%) of 244 in the HAIC combination group showed disease progression or died. Median PFS durations were 9.43 months in the HAIC combination group (95%CI: 8.07–10.7) and 10.97 months in the TACE-HAIC combination group (95%CI: 9.87–15.1), indicating no statistical significance (*p* = 0.104). Median OS durations were 19.3 months (95%CI: 18.1–25.9) and 26.8 months (95%CI: 18.2–NA) in the HAIC combination and TACE-HAIC combination groups, respectively, with no significant difference between the two groups (*p* = 0.53, **Figure 2**). ORRs were 59.9% and 63.0% in the HAIC combination and TACE-HAIC combination groups, respectively (*p* = 0.559); DCRs were 92.7% and 92.4%, respectively (*p* = 0.949, **Table 2**).

**Table 2:** Tumor responses evaluated by RECIST 1.1 before and after PSM.

Before PSM	Tumor Response	HAIC combined N=244(%)	TACE-HAIC combined N=119(%)	*P*
	CR	7(2.9)	0	0.240
	PR	139 (57.0)	75 (63.0)	
	SD	80 (32.8)	35 (29.4)	
	PD	18 (7.3)	9 (7.6)	
	ORR, (%)	59.9	63.0	0.559
	DCR, (%)	92.7	92.4	0.949
After PSM	Tumor Response (%)	HAIC combined N=143(%)	TACE-HAIC combined N=87(%)	*P*
	CR	3(2.1)	0	0.091
	PR	80(55.9)	56(64.4)	
	SD	47(32.9)	26(29.9)	
	PD	13(9.1)	5(5.7)	
	ORR, (%)	58.0	64.4	0.341
	DCR, (%)	90.9	94.3	0.360

CR, Complete Response; PR, Partial Response; SD, Stable Disease; PD, Progressive Disease; ORR, Objective Response Rate; DCR, Disease Control Rate.

#### After PSM

Median follow-up times were 24.07 (95%CI: 21.52-26.62) months in the HAIC combination group and 17.30 (95%CI: 13.18-21.43) months in the TACE-HAIC combination group (*p* < 0.001). Totally 31 (35.6%) of 87 cases in the TACE-HAIC combination group and 74 (51.7%) of 143 in the HAIC combination group showed disease progression or died. Median PFS durations were 9.07 months (95%CI: 7.60–10.1) and 11.17 months (95%CI: 9.93–16.6) in the HAIC combination and TACE-HAIC combination groups, respectively, indicating no statistical significance (*p* = 0.133). Median OS durations were 19.1 months in the HAIC combination group (95%CI: 18.1–24.9) and 26.8 months in the TACE-HAIC combination group (95%CI: 18.2–NA), also suggesting no statistical significance (*p* = 0.233, **Figure 2**). ORRs were 58.0% and 64.4% in the HAIC and TACE-HAIC combination groups (*p* = 0.341); DCRs were 90.9% and 94.3%, respectively (*p* = 0.360, **Table 2**).

### Subgroup analysis

**Figure 3:**
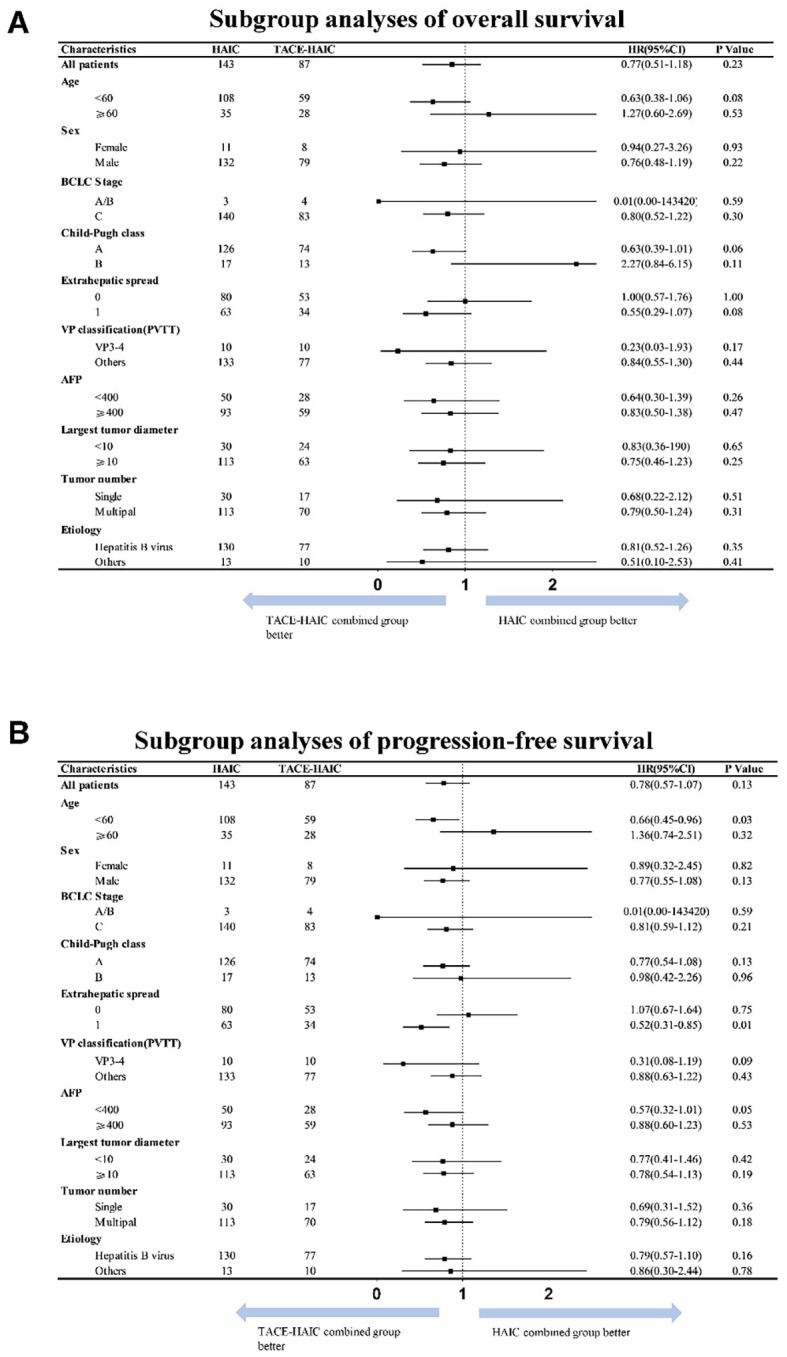
Subgroup analysis of progression-free survival **(A)** and overall survival **(B)** after matching.

After PSM, univariable and multivariable analyses by COX regression (**Table 3**) revealed tumor number (HR = 1.70, 95%CI: 1.13-2.56; *p* = 0.011) as the sole factor independently affecting PFS. Before PSM, VP classification (PVTT) independently predicted PFS. Univariable and multivariable analyses showed no statistically significant differences in OS between the two groups before and after matching (**Supplementary Table S1**, **Table 3**). Subgroup analysis also showed no statistically significant differences between the two groups (**Supplementary Figure S7**, **Figure 3**).

**Table 3:** Predictors of progression-free survival and overall survival after PSM.

	Univariable analysis			Multivariable analysis		
	HR	95%CI	*P*	HR	95%CI	*P*
PFS analyses						
Treatment	0.78	(0.57-1.08)	0.133			
Age(<60 vs. ≥60)	0.81	(0.58-1.13)	0.209			
Sex (Female vs. male)	0.87	(0.53-1.44)	0.589			
Etiology (HBV vs. others)	1.13	(0.68-1.86)	0.645			
BCLC Stage (A/8 vs. C)	4.83	(1.19-19.68)	0.028	2.68	(0.60-12.07)	0.199
Child-Pugh class (B vs. A)	1.52	(0.99-2.35)	0.059	1.57	(0.99-2.48)	0.053
Tumor diameter (cm)(<10 vs. ≥10)	1.24	(0.88-1.75)	0.210			
Tumor number (Single vs. Multiple)	1.93	(1.29-2.88)	0.001	1.70	(1.13-2.56)	0.011
VP classification (PVTT) (VP3-4 vs. others)	1.70	(0.94-3.07)	0.077	1.48	(0.77-2.83)	0.238
Extrahepatic spread (present vs. absent)	1.23	(0.91-1.66)	0.179			
OS analyses						
Treatment	0.77	(0.51-1.80)	0.233			
Age(<60 vs. ≥60)	1.08	(0.71-1.64)	0.709			
Sex (Female vs. male)	0.64	(0.35-1.17)	0.145			
Etiology (HBV vs. others)	1.25	(0.62-2.51)	0.541			
BCLC	2.14	(0.52-8.81)	0.292			
Child-Pugh class (B vs. A)	1.55	(0.91-2.64)	0.109			
Tumor diameter (cm)(<0 vs. ≥10)	1.29	(0.83-2.01)	0.259			
Tumor number (Single vs. Multiple)	1.87	(1.11-3.15)	0.019			
VP classification (PVTT) (VP3-4 vs. others)	1.62	(0.75-3.50)	0.220			
Extrahepatic spread (present vs. absent)	1.25	(0.85-1.84)	0.265			

The multivariable analysis includes the variables with p-value ≤0.1 from the univariable analysis. HR, hazard ratio; CI, confidence intervals; HBV, hepatitis B virus; BCLC, Barcelona Clinic Liver Cancer.

### Safety

The commonest adverse events (AEs) encompassed thrombocytopenia, hypertension, and increased AST (aspartate aminotransferase) and ALT (alanine aminotransferase) of any grade pre- and post-PSM. However, the TACE-HAIC combination group had elevated rates of fever, abdominal pain, ALT increase, and AST increase of any grade (*p* < 0.05), and grade 3–4 ALT and AST increases (*p* < 0.05) (**Supplementary Table S2**, **Table 4**).

## Discussion

Currently, combination therapies represent the trend in HCC management, and both targeted therapy plus immunotherapy and dual immunotherapy yield promising results. Additionally, TACE and HAIC constitute two commonly used interventional treatment methods (24). Could the combination of these two tools also confer survival benefits? To answer this question, the present study aimed to compare TACE-HAIC plus TKIs and ICIs to HAIC plus TKIs and ICIs for efficacy and safety to determine a more suitable local therapeutic strategy. However, in this study, HAIC and TACE-HAIC had no statistically significant differences in patients with high tumor burden when combined with TKIs and ICIs (median OS, 26.8 vs. 19.1 months, *p* = 0.233; median PFS, 11.17 vs. 9.01 months, *p* = 0.133). In a previous study, HAIC showed clear advantages over TACE in cases with tumors surpassing 7 cm (17). Similarly, HAIC demonstrated better efficacy versus TACE in cases with Vp4 involvement and hepatic arterioportal vein shunts (25). Considering the above findings, we hypothesize that HAIC may be highly important in treating patients with high tumor burden.

The reasons for no survival difference between the HAIC combination and TACE-HAIC combination groups are 1) tumor size (median tumor sizes in both groups all surpassed 10 cm [10.1 cm and 11.8 cm, respectively]) and 2) macrovascular invasion (most patients had macrovascular invasions, e.g., portal vein and hepatic vein invasion).

Regarding tumor size, previous evidence suggests a markedly lower rate of complete tumor response for large HCCs (>5 cm) versus smaller ones (25% vs. 64%) (26). A recent randomized phase III trial further highlighted the superiority of HAIC over TACE in individuals with large, unresectable HCCs (>7 cm), reporting OS of 23.1 months versus 16.1 months (27). Additionally, large HCCs require a high volume of embolic agents injected into the tumors, which may elevate the risk of liver function deterioration, post-embolization syndrome, and non-target embolization. However, whether chemotherapeutic drugs effectively enter the tumor following embolization remains unclear. As a result, TACE-HAIC shows no superior benefit over HAIC in patients with HCC lesions above 10 cm.

For macrovascular invasion, clinically, portal vein invasion often results in hepatic arterioportal vein fistula, which might induce embolic agents to migrate into the portal vein, increasing the risk of portal hypertension, esophagogastric varices rupture, and liver dysfunction. In case of hepatic vein invasion, small embolic agents can traverse the shunts to the inferior vena cava and pulmonary artery, inducing complications, e.g., dry cough from micropulmonary embolism. Furthermore, embolic agents can even enter the circulatory system via abnormal shunts, which may induce serious complications such as cerebral infarction. Therefore, in patients with macrovascular invasion, it is difficult for the lipiodol to deposit inside the tumor; instead, most of it is more likely lost via hepatic arteriovenous shunt. This may be the reason why TACE-HAIC is not effective for patients with PVTT (Vp3-4).

Examining previous studies on TACE-HAIC, several points worth discussing were identified. Firstly, the concept of TACE-HAIC needs clarification. How should the cases be defined if the tumor’s extrahepatic branches are embolized (e.g., internal mammary, renal capsular, and gastroduodenal arteries) and the microcatheter is retained in the hepatic artery to perfuse the main tumor-feeding vessels? As demonstrated in the **Supplementary Material** (Page 1-4, **Supplementary Figures S1–S4**), the “TACE-HAIC technique” should ensure that the embolization and perfusion areas exactly define the same area. All the individuals included in the TACE-HAIC group in this study met this criterion. Secondly, previous studies only pointed out the selection of chemotherapeutic drugs and the dosage of iodized oil embolization used in TACE-HAIC, not indicating how to deal with patients with hepatic arterioportal vein or hepatic arterio-hepatic vein shunts and whether sponge embolization should be given. Moreover, after complete embolization, when blood vessels are blocked, whether chemotherapeutic drugs can enter the tumor to the greatest extent is unknown. Additionally, unlike HAIC, the “TACE-HAIC technique” is hard to standardize, which makes its promotion difficult. Finally, the majority of existing reports assessing TACE-HAIC are single-arm studies or comparisons with TACE. Although TACE-HAIC has shown significantly improved survival benefits compared with TACE (14–20), we cannot rule out the possibility that some patients may benefit from HAIC. Currently, no study has compared TACE-HAIC and HAIC for efficacy.

Combining the current findings and previously reported data (21), there is substantial evidence to believe that HAIC plays as the pivotal component in the TACE-HAIC regimen, while TACE may contribute no therapeutic benefits but increase the incidence of AEs. An elevated rate of individuals in the TACE-HAIC combination group had fever, abdominal pain, and elevated ALT and AST of any level (*p* < 0.05), and grade 3–4 ALT and AST elevations (*p* < 0.05) (**Supplementary Table S2**, **Table 4**). On the other hand, use of embolic agents and chemotherapeutic drugs in TACE increases the economic burden on patients. From this standpoint, the TACE-HAIC regimen fails to demonstrate synergistic benefits.

**Table 4:** Treatment related adverse events after PSM.

Adverse event*	HAIC combined N=143(%)		TACE-HAIC combined N=87(%)		*P*	
	Any grade	Grade 3-4	Any grade	Grade 3-4	Any grade	Grade 3-4
Neutropenia	56 (39.8)	14 (9.8)	35 (40.2)	7 (8.0)	0.872	0.656
Anaemia	16 (11.2)	2 (1.4)	8 (9.2)	1 (1.1)	0.632	0.872
Fever	25 (17.5)	4 (2.8)	30(34.5)	5 (5.7)	0.003	0.271
Thrombocytopenia	88 (61.5)	25 (17.5)	46 (52.9)	20 (19.6)	0.196	0.307
Fatigue	35 (24.5)	5 (3.5)	25 (28.7)	2 (2.3)	0.476	0.608
Hypertension	62 (43.4)	11 (7.7)	32 (36.8)	6 (6.9)	0.325	0.823
Weight loss	46 (32.2)	4 (2.8)	29 (34.5)	5 (5.7)	0.716	0.263
Hypothyroidism	34 (23.8)	9 (6.3)	23 (26.4)	4 (4.6)	0.650	0.589
Vomiting	60 (42.0)	9 (6.3)	31 (35.6)	7 (8.0)	0.341	0.612
Diarrhea	14 (9.7)	2 (1.4)	10 (11.5)	4 (4.6)	0.669	0.140
Abdominal pain	31 (21.7)	4 (2.8)	48 (55.2)	9 (10.3)	<0.001	0.016
Elevated ALT	61 (42.7)	18 (12.6)	62 (71.3)	24 (27.6)	<0.001	0.004
Elevated AST	63 (44.1)	21 (14.7)	67 (77.0)	26 (29.9)	<0.001	0.006
Hyperbilirubinacemia	48 (33.6)	6 (4.2)	32 (36.8)	6 (6.9)	0.620	0.372
Hyboalbuminaemia	42 (29.4)	8 (5.6)	30 (34.5)	8 (9.2)	0.417	0.291
Immune-related hepatitis	4 (2.8)	1(0.7)	2 (2.3)	1 (1.1)	0.808	0.721
Immune-related pneumonitis	3 (2.1)	1(0.7)	2 (2.3)	1 (1.1)	0.929	0.721
Immune-related dermatitis	4 (2.8)	1(0.7)	5 (5.7)	0	0.263	0.434
Immune-related myocarditis	2 (1.4)	1(0.7)	2 (2.3)	0	0.613	0.434

* Listed are adverse events, as defined by the National Cancer Institute Common Terminology Criteria (version 5.0).

ALT, Alanine Aminotransferase; AST, Aspartate Transaminase.

The current study had multiple limitations. First, as a retrospective analysis, selection bias and absence of randomization are inevitable, even after PSM. Secondly, the follow-up period was short, with an insufficient number of OS events to draw definitive conclusions about the long-term therapeutic benefits, warranting long-term survival analyses. Thirdly, all cases were recruited from a single center, which limits data generalizability.

In light of these limitations, future prospective, multi-center randomized controlled trials (RCTs) are urgently needed to validate the current findings. Such RCTs should be designed with longer follow-up periods, comprehensive subgroup analyses, and biomarkers to fully compare the safety, efficacy, and clinical value of TACE-HAIC and HAIC in patients with high-tumor-burden HCC.

## Conclusion

Among HCC patients with high tumor burden, HAIC demonstrates comparable efficacy to TACE-HAIC both in combination with TKIs and ICIs. However, TACE-HAIC increases the financial burden on patients, induces further adverse events, and provides no significant improvements in PFS or OS compared with the HAIC procedure. Thus, we recommend HAIC as the preferred local treatment for HCC patients with high tumor burden, rather than the TACE-HAIC treatment.

## Data Availability

The raw data supporting the conclusions of this article will be made available by the authors, without undue reservation.
