# Correction: TACE-HAIC versus HAIC combined with TKIs and ICIs for hepatocellular carcinoma with a high tumor burden—a propensity-score matching comparative study

**DOI:** 10.3389/fimmu.2025.1769802

**Published:** 2026-01-02

**Authors:** Chunxue Wu, Yunlong Dong, Xinge Li, Wenbo Shao, Guangshun Wang, Huiyong Wu, Xu Chang

**Affiliations:** 1 Shandong Cancer Hospital and Institute, Shandong First Medical University and Shandong Academy of Medical Sciences, Jinan, China; 2 Department of Thoracic Surgery, Tianjin Baodi Hospital, Baodi Hospital Affiliated to Tianjin Medical University, Tianjin, China; 3 Central Hospital Affiliated to Shandong First Medical University, Jinan, China

**Keywords:** hepatocellular carcinoma, transarterial chemoembolization (TACE), hepatic arterial infusion chemotherapy (HAIC), combination therapy, high tumor burden

Affiliation “Shandong Cancer Hospital and Institute, Shandong First Medical University and Shandong Academy of Medical Sciences, Jinan, China” was erroneously given as “Shandong Tumor Hospital, Jinan, China”.

Affiliation “Department of Thoracic Surgery, Tianjin Baodi Hospital, Baodi Hospital Affiliated to Tianjin Medical University, Tianjin, China” was erroneously given as “Tianjin Baodi Hospital, Tianjin, China”.

The original version of this article has been updated.

