# Supplementary material for: TACE-HAIC versus HAIC combined with TKIs and ICIs for hepatocellular carcinoma with a high tumor burden—a propensity-score matching comparative study
Source: Front Immunol. 2025 Nov 24;16:1664756. doi: 10.3389/fimmu.2025.1664756 (PMC12682802; doi:10.3389/fimmu.2025.1664756)
Supplement: Supplementary file 1 [file DataSheet1.doc]

**SUPPLEMENTARY MATERIAL**

**Contents of Supplementary Appendix**

[**Figures S1-S4 Detailed definitions of the HAIC and TACE-HAIC combination groups. 2**](#__RefHeading___Toc211519529)

[**Figure S1. An example in the HAIC combination group. 2**](#__RefHeading___Toc211519530)

[**Figure S2. Another example in the HAIC combination group. 3**](#__RefHeading___Toc211519531)

[**Figure S3. An example in the TACE-HAIC combination group. 3**](#__RefHeading___Toc211519532)

[**Figure S4. An example of excluded cases. 4**](#__RefHeading___Toc211519533)

[**Figure S5. Jitter plot of the propensity score distribution after matching. 4**](#__RefHeading___Toc211519534)

[**Figure S6. Histogram of the propensity score distribution after matching. 5**](#__RefHeading___Toc211519535)

[**Figure S7. Subgroup analyses of progression-free survival (A) and overall survival (B) before matching 6**](#__RefHeading___Toc211519536)

[**Figure S8. Subgroup survival analysis of PD-1. 7**](#__RefHeading___Toc211519537)

[**Figure S9. Landmark survival analysis after PSM. 8**](#__RefHeading___Toc211519538)

[**Figure S10. Subgroup survival analysis of “massive tumor only” and “macrovascular invasion only” cohorts. 9**](#__RefHeading___Toc211519539)

[**Table S1 Baseline characteristics before and after PSM. 10**](#__RefHeading___Toc211519540)

[**Table S2. Predictors of progression-free survival and overall survival before PSM. 11**](#__RefHeading___Toc211519541)

[**Table S3. Treatment-related adverse events before PSM. 12**](#__RefHeading___Toc211519542)

# Figures S1-S4 Detailed definitions of the HAIC and TACE-HAIC combination groups.

The following images for 4 cases have been provided to give readers additional information about the definitions of the HAIC and TACE-HAIC groups.

## Figure S1. An example in the HAIC combination group.


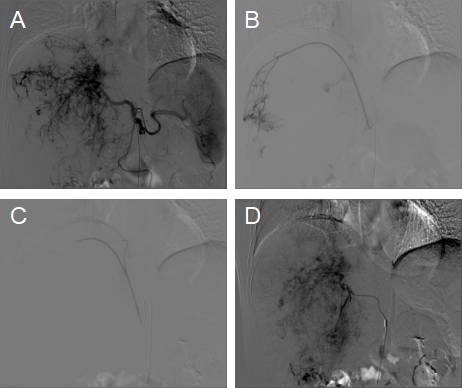


Celiac trunk angiography (**A**) and phrenic artery angiography (**B**) show obvious tumor staining. Gelatin sponge particles were used to embolize the inferior phrenic artery (**C**). A microcatheter was placed in the primary responsible vessel for hepatic perfusion (**D**).

We classified these cases into the HAIC combination group rather than the TACE-HAIC combination group. Similarly, cases with embolized liver's lateral branches (internal mammary artery, renal capsular artery, gastroduodenal artery) were also classified into the HAIC combination group.

## Figure S2. Another example in the HAIC combination group.


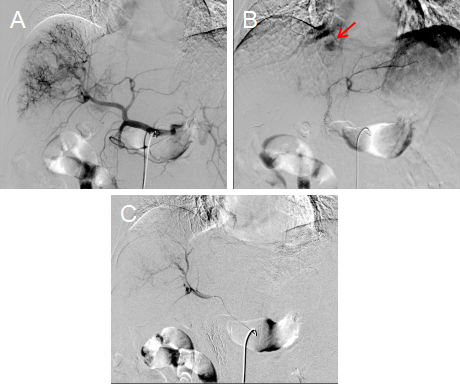


(A). Celiac trunk angiography showing obvious tumor staining. (B). Angiography of the left hepatic artery revealing obvious tumor staining. (C). Embolization of the tumor in the left lobe and placement of a microcatheter in the right hepatic artery.

These cases were classified into the HAIC combination group.

## Figure S3. An example in the TACE-HAIC combination group.


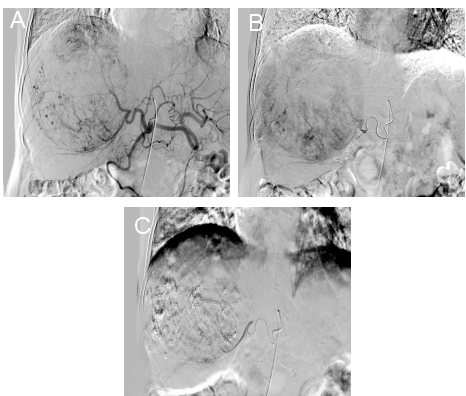


Celiac trunk angiography showing obvious tumor staining (A). After the microcatheter was super-selectively positioned, iodized oil and epirubicin were administered for embolization (B). The microcatheter was placed in the primary responsible blood vessel for hepatic perfusion (C).

We defined this protocol as the standard TACE-HAIC procedure, embolizing the main tumor-feeding vessels while leaving a microcatheter in the same main tumor-feeding vessels for hepatic perfusion. These patients were assigned to the TACE-HAIC combination group.

## Figure S4. An example of excluded cases.


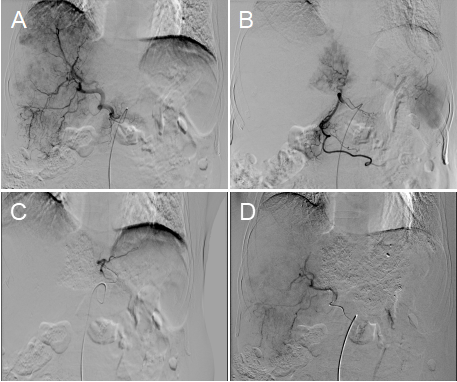


The superior mesenteric artery gives rise to a substitute right hepatic artery (A) and the celiac trunk gives rise to the middle hepatic artery (B); diffuse tumor staining can be seen. The left gastric artery gives rise to the accessory left hepatic artery (C) and obvious tumor staining can be observed. Embolization of the left gastric artery and the middle hepatic artery, and placement of a microcatheter in the right hepatic artery (D).

Such cases were not included in this study, for not meeting the criterion for the standard HAIC or TACE-HAIC procedure.

# Figure S5. Jitter plot of the propensity score distribution after matching.


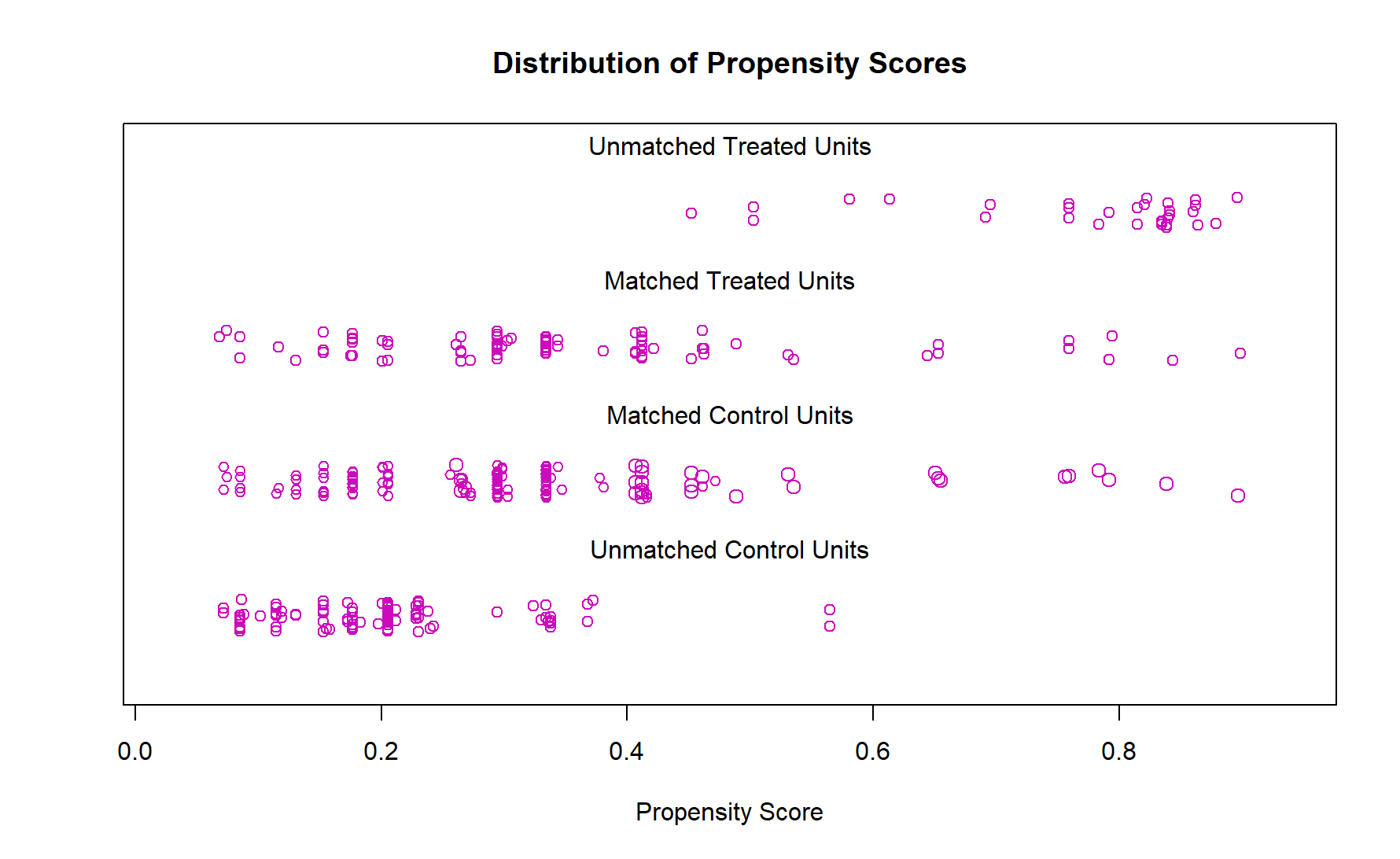


# Figure S6. Histogram of the propensity score distribution after matching.


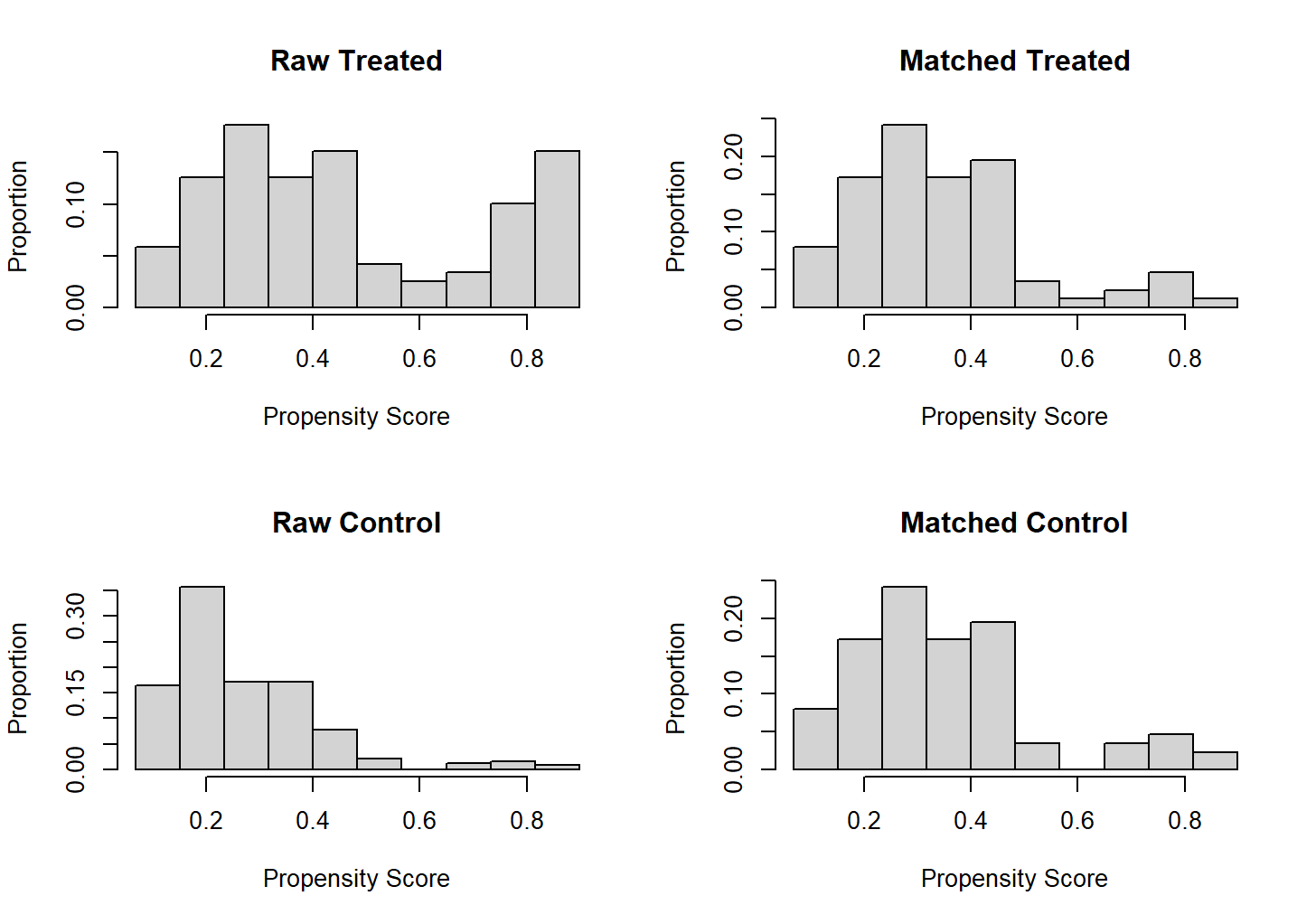


# Figure S7. Subgroup analyses of progression-free survival (A) and overall survival (B) before matching


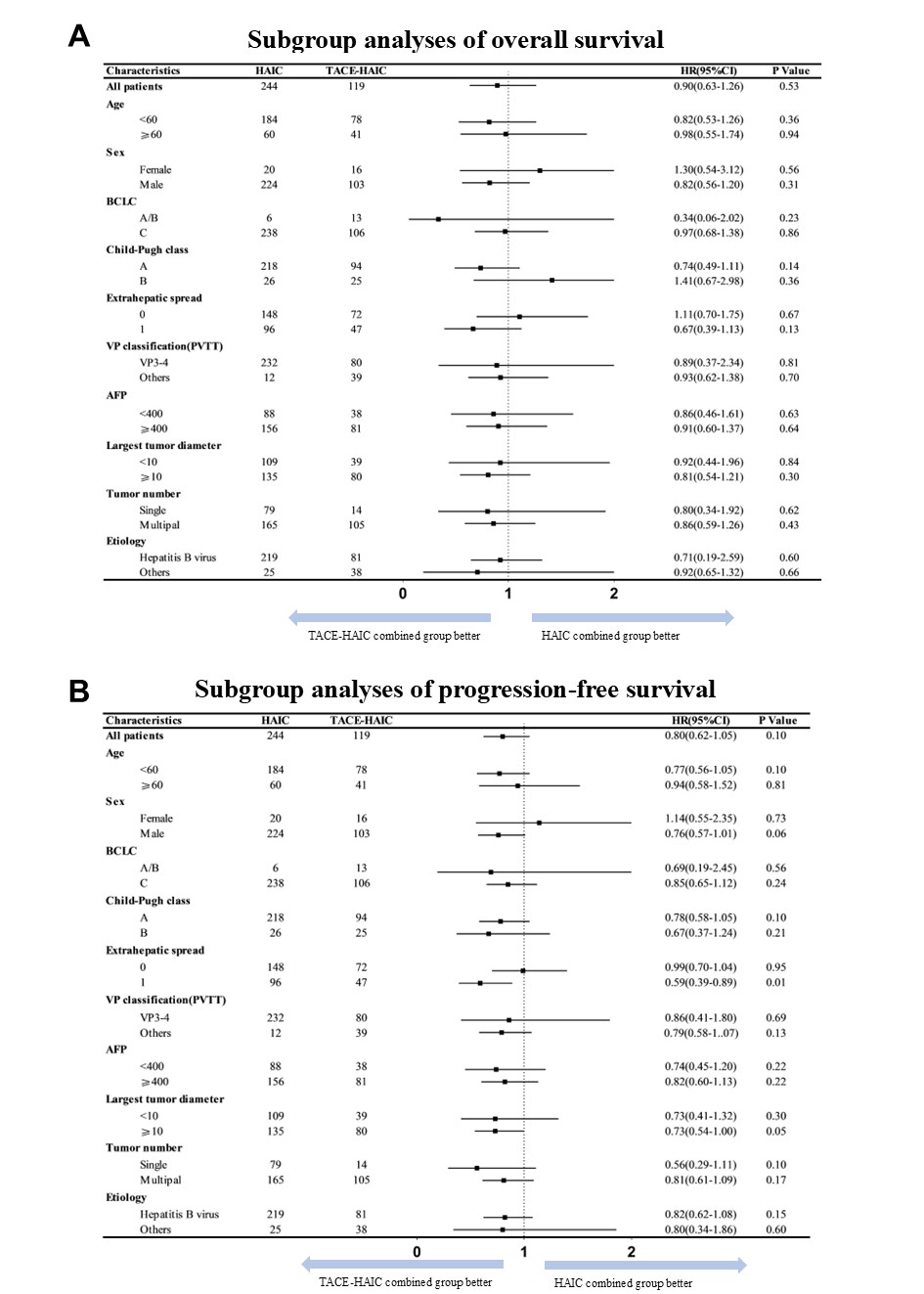


# Figure S8. Subgroup survival analysis of PD-1.





Kaplan-Meier analyses of progression-free survival (PFS) and overall survival (OS) according to different PD-1 inhibitors in patients receiving HAIC and TACE-HAIC treatment. The median PFS (**a**) of the camelizumba, sintilimab, and tisleizumab was 9.47(8.57-11.2) months, 10.10(8.90-15.2) months, and 9.93(7.03- NA) months in the whole cohort (*p*=0.66). The median OS (**b**) of the camelizumba, sintilimab, and tisleizumab was 20.3 (95%CI: 18.1-27.2) months. 24.6 (18.2- NA) months, and 17.1 (12.8- NA) months in the whole cohort (*p*=0.55). The median PFS (**c**) of the camelizumba, sintilimab, and tisleizumab was 9.3(7.3-12.3) months, 9.67(8.07-16.7) months, and 7.60(4.40-13.5) months in the HAIC combination group, respectively (*p*=0.62). The median OS (**d**) of the camelizumba, sintilimab, and tisleizumab was 19.1(16.2-26.2) months, 24.6(18.8- NA) months, and 12.8(10.5- NA) months in the HAIC combination group, respectively (*p*=0.18). The median PFS (**e**) of the camelizumba, sintilimab, and tisleizumab was 9.67 (8.47-13.6) months, 10.97(8.90- NA) months, and 21.9 (9.93- NA) months in the TACE-HAIC combination group, respectively (*p*=0.31). The median PFS (**f**) of the camelizumba, sintilimab, and tisleizumab was 26.8 (17.4- NA) months, 18.2 (11.7- NA) months, and NA in the TACE-HAIC combination group, respectively (*p*=074).

# Figure S9. Landmark survival analysis after PSM.


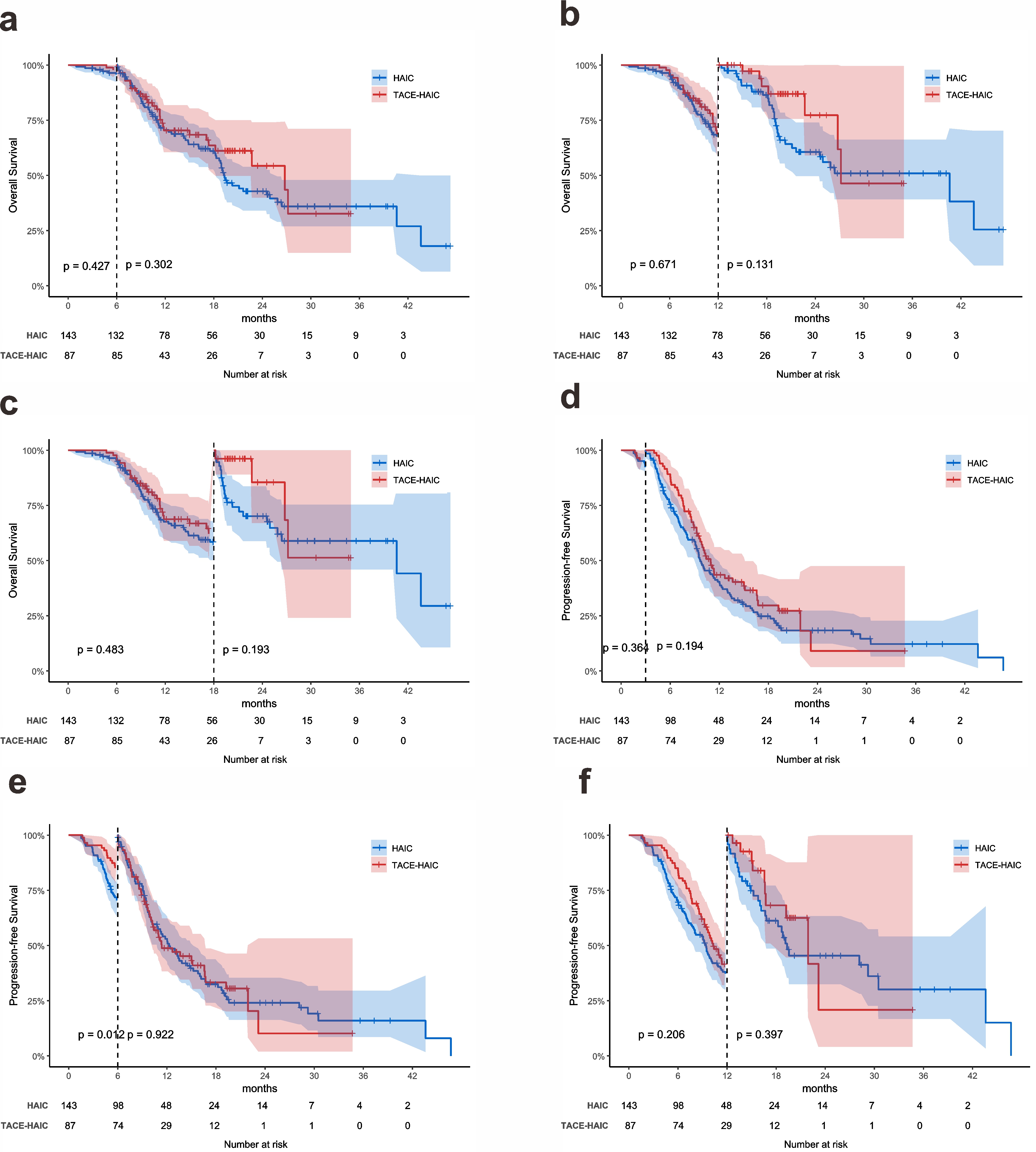


Kaplan-Meier curves of landmark analyses of overall survival (OS) at 6 months (a), 12 months (b), and 18 months (c). And Kaplan-Meier curves of landmark analyses of progression-free survival (PFS) at 3 months (d), 6 months (e), and 12 months (f).

# Figure S10. Subgroup survival analysis of “massive tumor only” and “macrovascular invasion only” cohorts.


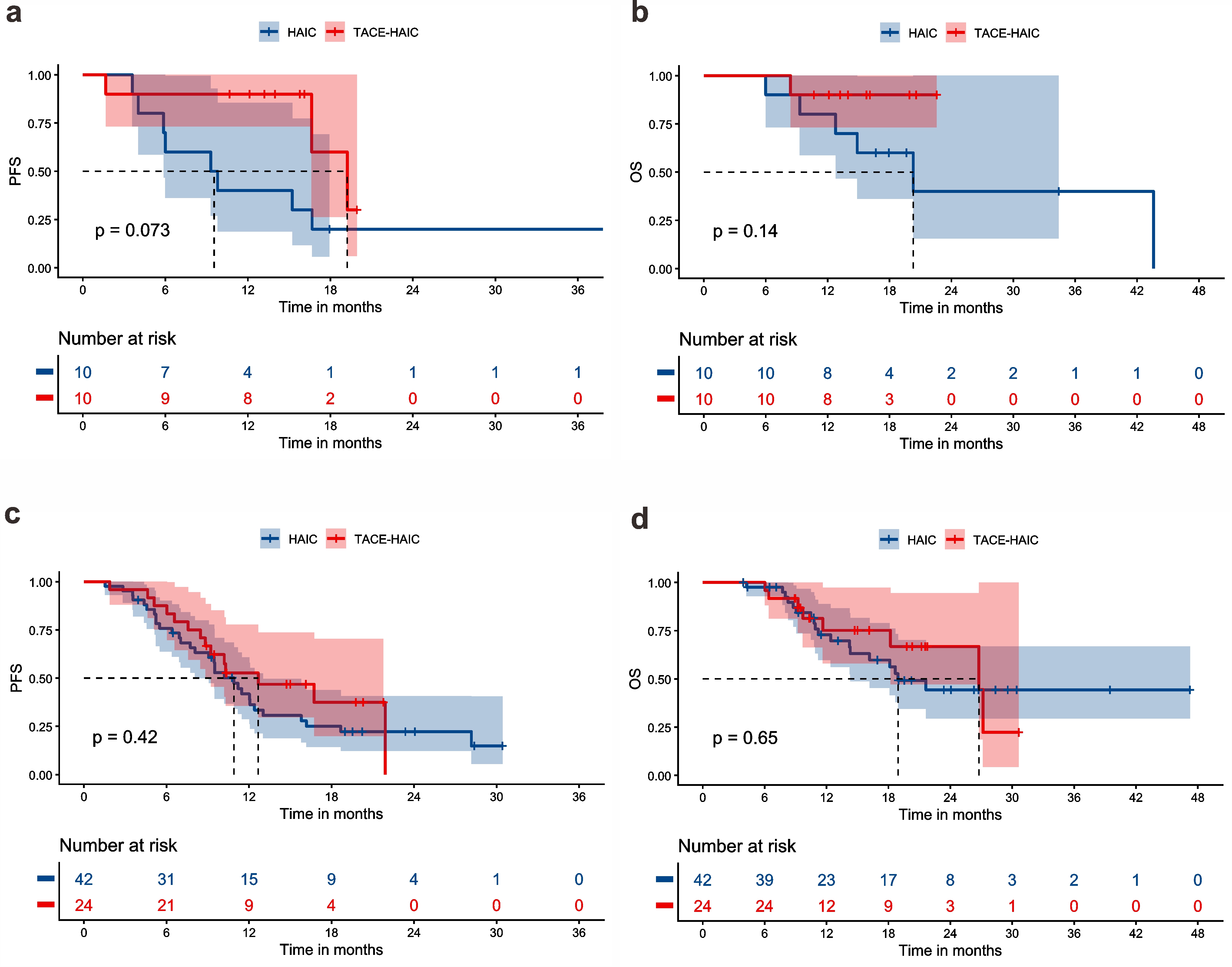


Kaplan-Meier analyses of progression-free survival (PFS) (a) and overall survival (OS) (b) “massive tumor only” cohort. And Kaplan-Meier analyses of PFS (c) and OS (d) of “macrovascular invasion only” cohort.

# Table S1 Baseline characteristics before and after PSM.

| **Characteristics** | **Before PSM** | | |  | | **After PSM** | | |
| --- | --- | --- | --- | --- | --- | --- | --- | --- |
|  | **HAIC combined**  **N=244(%)** | | **TACE-HAIC combined**  **N=119(%)** | **SMD** |  | **HAIC combined**  **N=143(%)** | **TACE-HAIC combined**  **N=87(%)** | **SMD** |
| **Age (years)**  <60  ≥60 | | 184(75.4)  60(24.6) | 78(65.5)  41(34.5) | 0.218 |  | 108(75.5)  35(24.5) | 59(67.8)  28(32.2) | 0.172 |
| **Gender** | |  |  | 0.17 |  |  |  | 0.054 |
| Male | | 224(91.8) | 103(86.6) |  |  | 132(92.3) | 79(90.8) |  |
| Female | | 20(8.2) | 16(13.4） |  |  | 11(7.7) | 8(9.2) |  |
| **Etiology** | |  |  | 0.049 |  |  |  | 0.079 |
| Hepatitis B virus | | 219(93.6) | 105(88.2) |  |  | 130(90.9) | 77(88.5) |  |
| Others | | 25(6.4) | 14(11.8) |  |  | 13(9.1) | 10(11.5) |  |
| **BCLC stage** | |  |  | 0.344 |  |  |  | 0.139 |
| A/ B | | 6(2.5) | 13(10.9) |  |  | 3(2.1) | 4(4.6) |  |
| C | | 238(97.5) | 106(89.1) |  |  | 140(97.9) | 83(95.4) |  |
| **Tumor diameter (cm)** | |  |  | 0.542 |  |  |  | 0.040 |
| <10 | | 109(44.7) | 24(20.2) |  |  | 42(29.4) | 24(27.6) |  |
| ≥10 | | 135(55.3) | 95(79.8) |  |  | 101(70.6) | 63(72.4) |  |
| **AFP (ng/ml)** | |  |  | 0.087 |  |  |  | 0.034 |
| <400 | | 88(36.1) | 38(31.9) |  |  | 50(35.0) | 28(32.2) |  |
| ≥400 | | 156(63.9) | 81(68.1) |  |  | 93(65.0) | 59(67.8) |  |
| **Tumor number** | |  |  | 0.345 |  |  |  | 0.036 |
| Single | | 79(32.4) | 21(17.6) |  |  | 30(21.0) | 17(19.5) |  |
| Multiple | | 165(67.6) | 98(82.4) |  |  | 113(79.0) | 70(80.5) |  |
| **Child-Pugh class** | |  |  | 0.286 |  |  |  | 0.090 |
| A | | 218(89.3) | 94(79.0) |  |  | 126(88.1) | 74(85.1) |  |
| B | | 26(10.7) | 25(21.0) |  |  | 17(11.9) | 13(14.9) |  |
| **VP classification (PVTT)** | |  |  | 0.762 |  |  |  | 0.156 |
| VP3-4 | | 232(95.1) | 80(67.2) |  |  | 133(93.0) | 77(88.5) |  |
| others | | 12(4.9) | 39(32.8) |  |  | 10(7.0) | 10(11.5) |  |
| **Extrahepatic spread** | |  |  | 0.003 |  |  |  | 0.101 |
| Present | | 96(39.3) | 47(39.5) |  |  | 63(44.1) | 34(39.1) |  |
| Absent | | 148(60.7) | 72(60.5) |  |  | 80(55.9) | 53(60.9) |  |
| **Tyrosine Kinase Inhibitors** | |  |  | 0.88 |  |  |  | 0.104 |
| sorafenib | | 30(12.3) | 12(10.1) |  |  | 20(14.0) | 10(11.5) |  |
| lenvatinib | | 139(57.0) | 83(69.7) |  |  | 82(57.3) | 59(67.8) |  |
| apatinib | | 75(30.7) | 24(20.2) |  |  | 41(28.7) | 18(20.7) |  |
| **Immune checkpoint inhibitors** | |  |  | 0.143 |  |  |  | 0.130 |
| camrelizumab | | 129(5.9) | 66(55.5) |  |  | 73(51.0) | 52(59.8) |  |
| sintilimab | | 44(18.0) | 23(19.3) |  |  | 32(22.4) | 17(19.5) |  |
| tislelizumab | | 47(19.3) | 22(18.5) |  |  | 24(16.8) | 13(14.9) |  |
| toripalimab | | 15(6.1) | 4(3.4) |  |  | 7(4.9) | 3(3.5) |  |
| othres | | 9(3.7) | 4(3.4) |  |  | 7(4.9) | 2(2.3) |  |
| **Totalbilirubin (mmol/L, mean±SD)** | | 20.3±14.7 | 20.9±11.6 | 0.043 |  | 20.7±12.4 | 20.5±10.8 | 0.001 |
| **Albumin (g/L, mean±SD )** | | 40.4±5.1 | 38.5±5.3 | 0.29 |  | 39.5±5.3 | 39.3±4.6 | 0.143 |
| PSM, Propensity Score Matching; SMD, Standardized Mean Difference; HAIC, Hepatic Arterial Infusion Chemotherapy; TACE, Transarterial Chemoembolization; BCLC, Barcelona Clinic Liver Cancer; AFP, Alpha-fetoprotein; PVTT, Portal Vein Tumor Thrombus. | | | | | | | | |

# Table S2. Predictors of progression-free survival and overall survival before PSM.

|  | **Univariable analysis** | | |  | **Multivariable analysis** | | |
| --- | --- | --- | --- | --- | --- | --- | --- |
|  | **HR** | **95%CI** | ***P*** |  | **HR** | **95%CI** | ***P*** |
| **PFS analyses** |  |  |  |  |  |  |  |
| Treatment | 0.80 | (0.62-1.05) | 0.104 |  |  |  |  |
| Age (<60 vs.≥60) | 0.89 | (0.68-1.16) | 0.382 |  |  |  |  |
| Sex (female vs. male) | 1.10 | (0.91-1.32) | 0.333 |  |  |  |  |
| Etiology (HBV vs. others) | 1.24 | (0.83-1.84) | 0.299 |  |  |  |  |
| BCLC stage (A/8 vs. C) | 1.87 | (1.02-3.42) | 0.042 |  | 1.73 | (0.93-3.21) | 0.083 |
| Child-Pugh class (B vs. A) | 1.44 | (1.04-2.01) | 0.029 |  | 1.30 | (0.93-1.83) | 0.124 |
| Tumor diameter (cm) (<10 vs. ≥10) | 1.28 | (0.99-1.64) | 0.056 |  | 1.23 | (0.95-1.60) | 0.122 |
| Tumor number (single vs. multiple) | 0.81 | (0.71-0.94) | 0.004 |  | 0.84 | (0.73-0.97) | 0.015 |
| VP classification (PVTT) (VP3-4 vs. others) | 1.09 | (0.77-1.54) | 0.625 |  |  |  |  |
| Extrahepatic spread (present vs. absent) | 1.40 | (1.10-1.78) | 0.006 |  | 1.27 | (0.99-1.62) | 0.061 |
| **OS analyses** |  |  |  |  |  |  |  |
| Treatment | 0.90 | (0.63-1.26) | 0.530 |  |  |  |  |
| Age (<60 vs.≥60) | 1.39 | (1.00-1.92) | 0.049 |  | 1.39 | (0.99-1.96) | 0.058 |
| Sex (female vs. male) | 1.25 | (1.00-1.56) | 0.055 |  | 1.14 | (0.90-1.44) | 0.268 |
| Etiology (HBV vs. others) | 1.28 | (0.74-2.22) | 0.377 |  |  |  |  |
| BCLC stage (A/8 vs. C) | 1.59 | (0.70-3.60) | 0.265 |  |  |  |  |
| Child-Pugh class (B vs. A) | 1.53 | (1.02-2.30) | 0.040 |  | 1.42 | (0.94-2.14) | 0.097 |
| Tumor diameter (cm) (<10 vs. ≥10) | 1.28 | (0.93-1.77) | 0.132 |  |  |  |  |
| Tumor number (single vs. multiple) | 0.83 | (0.69-0.99) | 0.039 |  | 0.75 | (0.71-1.01) | 0.066 |
| VP classification (PVTT) (VP3-4 vs. others) | 1.13 | (0.71-1.78) | 0.615 |  |  |  |  |
| Extrahepatic spread (present vs. absent) | 1.34 | (0.98-1.83) | 0.064 |  | 1.32 | (0.96-1.81) | 0.089 |
| The multivariable analysis includes variables with p<0.1 from the univariable analysis. HR, hazard ratio; CI, confidence interval; HBV, hepatitis B virus; BCLC, Barcelona Clinic Liver Cancer. | | | | | | | |

# Table S3. Treatment-related adverse events before PSM.

| **Adverse event*** | **HAIC combined**  **N=244(%)** | |  | **TACE-HAIC combined**  **N=119(%)** | |  | ***P*** | |
| --- | --- | --- | --- | --- | --- | --- | --- | --- |
|  | **Any grade** | **Grade 3-4** |  | **Any grade** | **Grade 3-4** |  | **Any grade** | **Grade 3-4** |
| Neutropenia | 94 (38.5) | 26 (10.7) |  | 48 (40.3) | 10 (8.4) |  | 0.740 | 0.5 |
| Anemia | 31 (12.7) | 5 (2.0) |  | 11 (9.2) | 3 (2.5) |  | 0.333 | 0.774 |
| Fever | 43 (17.6) | 10 (4.1) |  | 40(33.6) | 8 (6.7) |  | 0.001 | 0.280 |
| Thrombocytopenia | 152 (62.3) | 61 (24.9) |  | 68 (57.1) | 24 (20.2) |  | 0.346 | 0.317 |
| Fatigue | 73 (29.9) | 11 (4.5) |  | 36 (30.3) | 4 (3.4) |  | 0.948 | 0.606 |
| Hypertension | 114 (46.7) | 24 (9.8) |  | 50 (42) | 9 (7.6) |  | 0.398 | 0.479 |
| Weight loss | 78 (32) | 12 (4.9) |  | 39 (32.8) | 8 (6.77) |  | 0.877 | 0.479 |
| Hypothyroidism | 64 (26.2) | 18 (7.4) |  | 32 (26.9) | 7 (5.9) |  | 0.893 | 0.598 |
| Vomiting | 89 (36.5) | 18 (7.4) |  | 43 (36.1) | 10 (8.4) |  | 0.949 | 0.731 |
| Diarrhea | 25 (10.2) | 5 (2.0) |  | 14 (11.8) | 5 (4.2) |  | 0.661 | 0.240 |
| Abdominal pain | 69 (28.3) | 12 (4.9) |  | 68 (57.1) | 12 (10.1) |  | <0.001 | 0.063 |
| Elevated ALT | 105 (43) | 38 (15.6) |  | 86 (72.3) | 30 (25.2) |  | <0.001 | 0.027 |
| Elevated AST | 108 (44.3) | 40 (16.4) |  | 88 (73.9) | 32 (26.9) |  | <0.001 | 0.019 |
| Hyperbilirubinemia | 81 (33.2) | 17 (7.0) |  | 46 (38.7) | 10 (8.4) |  | 0.306 | 0.624 |
| Hypoalbuminemia | 78 (32.0) | 20 (8.2) |  | 47 (39.5) | 15 (12.6) |  | 0.156 | 0.182 |
| Immune-related hepatitis | 12 (4.9) | 4 (1.6) |  | 4 (3.4) | 1 (0.8) |  | 0.498 | 0.540 |
| Immune-related pneumonitis | 7 (2.9) | 3 (1.2) |  | 4 (3.4) | 2 (1.7) |  | 0.797 | 0.729 |
| Immune-related dermatitis | 10 (4.1) | 2 (0.8) |  | 6 (5.0) | 0 |  | 0.681 | 0.322 |
| Immune-related myocarditis | 7 (2.9) | 2 (0.8) |  | 3 (2.5) | 0 |  | 0.849 | 0.322 |
| *: Listed are adverse events as defined by the National Cancer Institute Common Terminology Criteria (version 5.0).  ALT, Alanine Aminotransferase; AST, Aspartate Transaminase. | | | | | | | | |
